# Association of Acute Headache of COVID-19 and Anxiety/Depression Symptoms in Adults Undergoing Post-COVID-19 Rehabilitation

**DOI:** 10.3390/jcm11175002

**Published:** 2022-08-26

**Authors:** Justyna Mazurek, Błażej Cieślik, Patryk Szary, Sebastian Rutkowski, Jan Szczegielniak, Joanna Szczepańska-Gieracha, Robert Gajda

**Affiliations:** 1 University Rehabilitation Centre, Wroclaw Medical University, 50-367 Wroclaw, Poland; 2 Department of Kinesiology and Health Prevention, Jan Dlugosz University in Czestochowa, 42-200 Czestochowa, Poland; 3 Faculty of Physiotherapy, Wroclaw University of Health and Sport Sciences, 51-612 Wroclaw, Poland; 4 Faculty of Physical Education and Physiotherapy, Opole University of Technology, 45-758 Opole, Poland

**Keywords:** COVID-19, headache, pain, depression, anxiety, stress, quality of life, long-term complications

## Abstract

As a common non-respiratory symptom of COVID-19, headache should not be overlooked, and its characteristics should be recorded with scrutiny. Identifying risk factors associated with post-COVID headache will ensure immediate action and counseling for this population of patients. Therefore, the study aimed to investigate the relationship between headache and psychological state (stress level, depression, and anxiety symptoms) in adults undergoing post-COVID-19 rehabilitation. In addition, we used mediation analysis to evaluate the mediation effect of psychological variables in the relationship between headache and quality of life. This cross-sectional study included 147 patients undergoing post-COVID-19 rehabilitation at the Public Hospital in Poland (64 males, 83 females, with mean age of 56.97 years). Psychological parameters were evaluated using the Hospital Anxiety and Depression Scale (HADS), the Perceived Stress Scale (PSS-10), and the brief World Health Organization Quality of Life Scale (WHOQOL-BRIEF). Additionally, all participants completed a questionnaire related to COVID-19 symptoms and their severity, the place of COVID-19 treatment, and the need for oxygen therapy during hospitalization. Of all participants, 65% experienced headache during COVID-19. Of the participants with headache, there were significantly more females in this group (69% vs. 31%), and they were significantly younger (mean age 55.47 vs. 59.78 years). Participants with headache had a 27% higher HADS-D score, a 21% higher HADS-A score, and a 13% higher PSS-10 score. Moreover, gender and headache were found to be important predictor variables for total HADS and HADS-D, accounting for 11% and 7%, respectively. Mediation analysis has shown that the tested psychological variables mediated 39–68% of the total effect of headache influence on WHOQOL domains. In conclusion, our study demonstrated several relationships between headache that occurred during COVID-19 and symptoms of depression, anxiety, and perceived stress level during post-COVID rehabilitation also in the context of quality of life. Our results show that patients who experienced headaches during COVID-19 are at high risk of developing anxiety-depressive symptoms later. Female gender is associated with a higher prevalence of headache during COVID-19.

## 1. Introduction

Patients with coronavirus disease 2019 (COVID-19) can experience a wide range of clinical manifestations, from no symptoms to critical illness. Initially, COVID-19 was described as a respiratory disease; however, when the number of cases began to increase, there were reports that other organs and systems were also affected by the disease as well [1].

Headache, depending on the study, is described by 14 to 70% of COVID-19 patients, which means that there is a considerable disparity in the reported prevalence [2]. According to the Centers for Disease Control and Prevention (CDC), it is the most common neurological symptom, experienced by 14.8% of hospitalized patients, reaching a prevalence of 22.7% of patients aged 18 to 49 years [3]. In a meta-analysis involving 6486 patients included in 21 studies, in which the prevalence ranged from 3.5–34% [4], the prevalence of headache was calculated to be 10.9%. In most studies, the incidence of headache in patients with COVID-19 is approximately 12–13% [5]. A recent survey found that headache was the most common neurological symptom (61.9%) [6] associated with COVID-19 observed by the neurologist. The true prevalence and phenotype of headache in COVID-19 remain unclear, as most of the available studies are series of hospitalized patients.

It remains unknown whether headache can be misdiagnosed as a primary headache disorder in COVID-19 patients treated in outpatient or rehabilitation settings. Furthermore, it is not known whether headache may be a significant clinical symptom predictive of the course of COVID-19 itself, which could guide clinicians in their evaluations of patients with COVID-19 in future waves of the pandemic. Much attention has been paid to clarifying the criteria for headache attributed to systemic infection. Headache is usually diffuse and bilateral, but in some cases, it may be frontotemporal or occipital with associated retroocular pain. The severity of headache varies and can be exacerbated by coughing, straining, or head movement [7]. 

One of the most common non-respiratory symptoms of COVID-19 is headache [1]. The classification of headache phenotypes associated with COVID-19 headache is as follows: (1) migraine phenotype, (2) tension-type headache phenotype and (3) cough headache phenotype [8]. Although the Centers for Disease Control and Prevention [9] have included headache as one of the main symptoms of COVID-19, currently there is no better definition of headache related to COVID-19 and its characteristics, and there are no definite data on its evolution. The third edition of the International Classification of Headache Disorders (ICHD-3) [10] includes headache attributed to systemic viral infection and, although it is commonly reported [11], there are no specific data available. 

Many recent studies add to a growing body of evidence suggesting the importance of considering potential neuropsychiatric sequelae of COVID-19 infection [12,13]. After acute infection with severe acute respiratory syndrome coronavirus 2 (SARS-CoV-2), a some of individuals experience persistent symptoms involving mood, sleep, anxiety, and fatigue, which may contribute to markedly elevated rates of major depressive disorder [14]. Perlis and colleagues found a link between headaches during COVID-19 and a higher risk of depression [15]. 

In a large cross-sectional European study, Lampl et al. confirmed that depression and especially anxiety are comorbid more than is expected by chance with headache in patients with migraine [16]. Importantly, medication overuse headache is also associated with depression, anxiety, and stress [17].

Headache in the acute phase of SARS-CoV-2 infection was also associated with an increased incidence of headache and fatigue as long-term symptoms following COVID [18]. It therefore seems that monitoring headache can help identify patients at risk of developing long-term symptoms after COVID, including fatigue, depression and anxiety symptoms.

Long-term complications arise from this multifaceted picture. It is also possible that COVID-19 as a headache trigger may cause chronic headache disorders, such as a new persistent headache. Therefore, it will become more important to closely follow up COVID-19 patients [5]. There are patients whose recovery after the acute phase is not complete and who experience ‘post-COVID syndrome’, that is, persistent symptoms and/or delayed or long-term complications of COVID-19 [19]. These symptoms may include memory impairment, insomnia, fatigue, dizziness, etc. Headache is also a symptom that occurs in some patients after COVID-19, and consults for persistent headache attributed to COVID-19, frequently referred to as ‘post-COVID headache’, are presumably becoming more common in clinical practice [20].

Headache attributed to COVID-19 can be persistent and disabling in certain cases, even in patients with no prior history of headache [21]. In the first 180 days, the time course of post-COVID headache appears to be stable, but longitudinal studies are necessary [22]. The predictors of an increase in the intensity of headache [7] attributed to COVID-19 include fever, dehydration, and gender. The severity of COVID-19-related headache is greater in patients with primary headache group disorders [23].

As a common non-respiratory symptom of COVID-19, headache should not be overlooked, and its characteristics should be recorded with scrutiny. Identifying risk factors associated with post-COVID headache will ensure immediate action and counseling for this population of patients. Therefore, the study aimed to investigate the relationship between headache and psychological state (stress level, depression, and anxiety symptoms) in adults undergoing post-COVID-19 rehabilitation.

## 2. Materials and Methods

### 2.1. Study Settings and Participants

This cross-sectional study included 147 patients who survived COVID-19 (64 females, 83 males, with a mean age of 56.97, SD 9.86), the average time elapsed between onset and commencement of post-COVID rehabilitation was 19.13 (SD 5.61) weeks. Included patients were undergoing post-COVID-19 rehabilitation at the Public Hospital in Glucholazy (Glucholazy, Poland). Dyspnea and fatigue, which hindered everyday functioning, was the main reason for admitting the patient to the ward and undergoing pulmonary and cardiovascular rehabilitation. Exclusion criteria were limited to patients under 18 years of age, a cognitive impairment that prevents psychological evaluation, any serious psychiatric disorders (e.g., bipolar disorder, major depression, and schizophrenia), and initiation of psychiatric or psychological treatment during the course of the study. Data were collected from patients admitted to the rehabilitation ward between July and September 2021. The research was in accordance with all relevant national regulations, institutional policies, and tenets of the Declaration of Helsinki. All study procedures were approved by the Institutional Review Board of the Wroclaw University of Health and Sport Sciences (Wroclaw, Poland) (nr 13/2021). No compensation was offered to the participants. Written informed consent was obtained from all individuals included in the study.

### 2.2. Outcome Measures

#### 2.2.1. Survey

All participants completed a questionnaire related to their sociodemographics, such as age, sex, education, marital status, and employment. They also responded to questions regarding their current health and health before COVID-19 (good, neither good nor bad, bad), physical activity before COVID-19 (yes/no), and what the symptoms of COVID-19 were and their severity. Additionally, questions were asked about comorbidities, the place of COVID-19 treatment, and the need for oxygen therapy during hospitalization.

#### 2.2.2. Depression and Anxiety Symptoms

To assess the severity of symptoms of depression and anxiety, the Hospital Anxiety and Depression Scale (HADS) was used. It is a 14-item scale scoring from 0 to 3 for each item. Seven items related to anxiety (HADS-A), while the remaining seven related to depression (HADS-D). The global score ranged from 0 to 42, with a cut-off point of 8/21 for anxiety and 8/21 for depression. The higher the score, the greater the anxiety or depression symptoms. According to the authors, the Cronbach alpha ranges from 0.78 to 0.93 for HADS-A and from 0.82 to 0.90 for HADS-D, and the test/retest correlations were *r* = 0.80 [24].

#### 2.2.3. Assessment of Stress Level

The Perceived Stress Scale (PSS-10) was used to measure psychological distress. It contains 10 questions on a five-point scale from 0 to 4 [25]. Participants are asked to rate their stress levels during the past month. The higher the score, the greater the feeling of stress. The scale demonstrated a satisfactory internal consistency (Cronbach’s alpha = 0.69).

#### 2.2.4. Quality of Life Assessment

Quality of life was assessed using the short version of the World Health Organization Quality of Life (WHOQOL-BREF). This questionnaire assesses quality of life in four domains: physical health, psychological, social relationships, and environmental. It contains 26 questions with answers arranged on a five-point Likert scale. WHOQOL-BREF is a cross-culturally valid assessment of quality of life, with an internal consistency alpha varying in each domain from 0.68 to 0.82 [26].

### 2.3. Data Analysis

Data were analyzed using SPSS 25.0 software (IBM Corp, Washington, DC, USA) and JASP 0.16.1 software (University of Amsterdam, Amsterdam, The Netherlands). Continuous variables are presented as means and standard deviations (SDs), and the categorical responses are presented as frequencies and percentages. Multiple linear regression (stepwise) was used to identify the association between headache during COVID-19 and depression and anxiety symptoms and stress levels. Prior to the multiple linear regression analysis, the assumption of a linear relationship (using the point biserial correlation coefficient) between the outcome variable and the independent variables was tested (variable encoding: 0—headache, 1—without headache). Three variables (stress level, depression, and anxiety symptoms) were tested as possible mediators of the relations between headache and quality of life outcomes. Mediators were tested by calculating bias-corrected 95% CI using bootstrapping (5000) with the JASP software. The variables were constructed as independent single mediator models. These models were based on different domains of quality of life, that is, physical health, psychological, social relationships, and environmental health. The results were presented as the effect size of the total, direct, and indirect effects. A significance level of α < 0.05 was established.

## 3. Results

### 3.1. Participants Characteristics

The ages of the participants were between 29 and 81 (mean 56.97, SD 9.86). The majority of respondents possessed higher education (46%), were professionally active (65%), and were married (70%). Of all participants, 65% experienced headache during COVID-19. There were significantly more females in this group (69% vs. 31%, *p* < 0.001), they were significantly younger (mean age 55.47 vs. 59.78 years, *p* = 0.01), body mass (82.59 vs. 86.59 kg, *p* = 0.02). Most of the study participants were physically active prior to COVID-19 (70%) and described their health as good (68%). However, most people (67%) described their current health as ‘neither good nor bad.’

Participants with headache were less frequently hospitalized for symptoms related to COVID-19 (44% vs. 76%, *p* < 0.001) and less often used oxygen therapy (48% vs. 68%, *p* = 0.01). Taking into account other symptoms, participants with headache experienced significantly less abdominal pain (67% vs. 88%, *p* = 0.006), muscle pain (14% vs. 49%, *p* < 0.001) and loss of smell and taste (30% vs. 55%, *p* = 0.003). Table 1 illustrates the detailed characteristics of the participants. 

### 3.2. Between-Group Comparison

Taking into account the results of the intergroup comparison, significant statistical differences were found in all variables examined, except for WHOQOL social relationships domain (*p* = 0.051). Participants with headache had a 27% higher level of HADS-D (*p* = 0.001), a 21% higher level of HADS-A (*p* < 0.001), and a 13% higher level of PSS-10 (*p* = 0.28). Participants with headache were also characterized by statistically significantly lower scores for the quality-of-life domains (Table 2). They obtained scores of 12, 6, and 8% lower for the domains of physical health (*p* = 0.016), psychological (*p* = 0.018), and environmental (*p* = 0.002), respectively. All statistically significant differences were within the moderate effect size range (Cohens d ranged from 0.43 to 0.61).

### 3.3. Correlations in the Data Set

**Figure 1:**
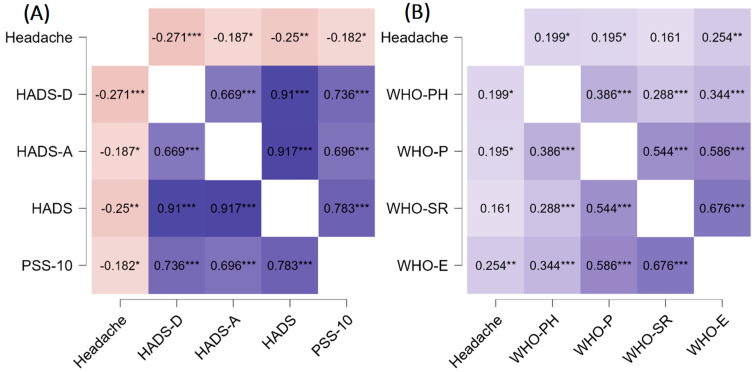
Correlation results with heatmap for psychological parameters (**A**) and quality of life (**B**). * *p* < 0.05; ** *p* < 0.01; *** *p* < 0.001. HADS: Hospital Anxiety (A) and Depression (D) scale; PSS-10: Perceived Stress Scale; WHO-PH: Physical health; WHO-P: Psychological; WHO-SR: Social relationships; WHO-E: Environmental.

Figure 1 represents headache correlation heatmap with psychological parameters and domains of quality of life. Analyses found that headache experienced during COVID-19 was strongly correlated with all post-COVID psychological parameters examined. The correlation coefficient ranged from −0.18 to −0.27, with the highest results for HADS-D. In terms of quality of life, headache during COVID was associated with worse outcomes of post-COVID physical health (r = 0.19), social relationships (r = 0.19), and environmental (r = 0.25) in WHOQOL-BREF domains.

### 3.4. Predictors

In order to examine how headache experienced during COVID-19 can explain a statistically significant amount of variance in post-COVID depression, anxiety, and stress level, stepwise multiple regression was used. Headache, gender, and age were included as a predictor. For total HADS and HADS-D, gender and headache were found to be important predictor variables, accounting for 11% and 7% of the variance in these models (*p* = 0.01 and *p* = 0.002, respectively) (Table 3). Among the variables included in the anxiety model (HADS-A), headache was revealed to be a significant predictor. This model accounted for 4% of the anxiety variance (*p* = 0.02). Taking into account the stress level (PSS-10), it was found that gender was a significant predictor (*p* = 0.001).

### 3.5. Mediation Analysis

**Figure 2:**
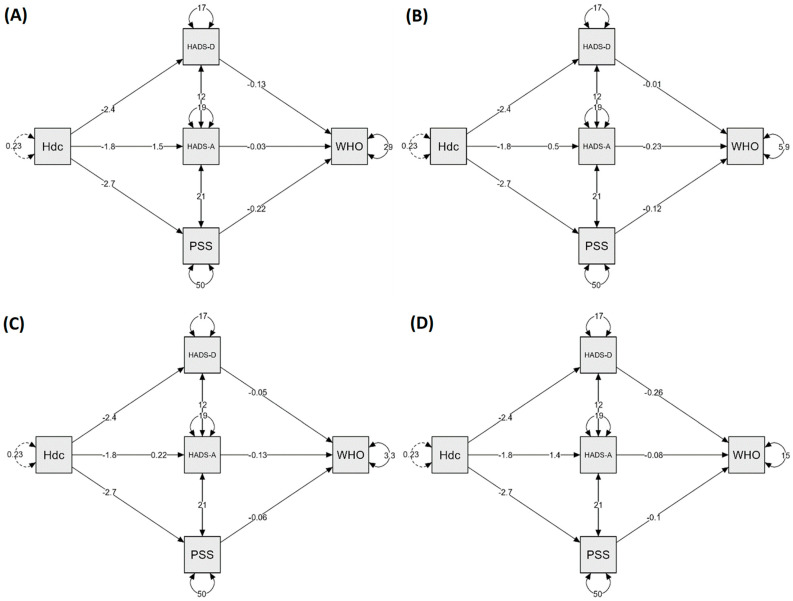
Mediation models for WHO domains: physical health (**A**), psychological (**B**), social relationships (**C**), and environmental (**D**). Hdc: Headache; HADS: Hospital Anxiety (A) and Depression (D) scale; PSS-10: Perceived Stress Scale.

Three variables (stress level, depression and anxiety symptoms) were tested as possible mediators of the relations between headache during COVID-19 and post-COVID quality of life outcomes (Figure 2). In all the models tested, the direct effect was not statistically significant. In turn, the indirect effect was significant, which resulted in the significance of the total effect. The tested variables mediated 39%, 60%, 68%, and 43% of the total effect of physical health, psychological, social relationships, and environmental WHOQOL domains, respectively (Table 4). 

## 4. Discussion

COVID-19 is a systemic inflammation that affects all age groups, with a high mortality rate and severe adverse outcomes. It involves the nervous system, blood vessels, lung, heart, liver, gastrointestinal system, kidney, eyes, and other organs [27]. It is important to keep in mind that as the COVID-19 pandemic rapidly sweeps across the world, it induces a considerable degree of negative economic and psychosocial consequences that can contribute to poor mental health. 

COVID-19-related headache was a commonly reported symptom in many studies, but there was a great diversity in its frequency, severity, character, and duration, with a prevalence of headache ranging from 3.5 to 34% [4]. There is no consensus on how COVID-19 affects the nervous system, and the mechanisms involved in COVID-19 headache are also unclear. Psychological and neurological symptoms after COVID-19 may be explained by different pathophysiological bases such as direct neuroinvasion with damage to the neuronal pathway, indirect effects mediated by hypoxia, hypertension, coagulopathy, and cytokine storm in the CNS, worsening of preexisting brain diseases or new disorder [28]. 

In the present study, 65.3% of the participants reported headache during COVID-19. The frequency of headache observed in this study was much higher than that reported in most studies. We have to remember that the study included people who suffered long-term complications after COVID and therefore entered rehabilitation. 

Previously published surveys showed increased symptoms of depression, anxiety, and stress-related to COVID-19, as a possible result of psychosocial stressors such as fear of the disease, loss of life, and economic issues [29,30,31]. Numerous studies in the general population have consistently shown that headache is more prevalent in women than in men. The most important risk factors for headache include overuse of acute migraine medications, ineffective acute treatment, obesity, depression, stressful life events, age, and low education level [32,33].

None of the results of the published surveys evaluated the relationship between headache as a somatic symptom related to the COVID-19 pandemic and psychological parameters or quality of life for long-COVID observation. It seems to be important to find a simple method to identify the group at high risk for developing psychiatric disorders and to provide early preventive measures or treatment to avoid further consequences. To our knowledge, this is the first study to investigate whether COVID-19 with a coexisting headache has a significant negative impact on patient psychological parameters and quality of life for 6 months after an acute COVID-19 infection.

Our findings present a comparison of the results of sociodemographic and clinical data between two groups of people undergoing post-COVID rehabilitation: 1/with acute comorbid headache during the disease and 2/without this symptom. All examined psychological parameters differed significantly to the disadvantage of the headache group, which means that people who reported headache during COVID-19 infection had worse post-COVID emotional state: a higher level of anxiety and depression, a higher level of stress and, at the same time, lower quality of life. The largest difference was observed for depression (*r* = −0.27). 

In order to answer the question: which variable may predispose to lowering or increasing the level of depression, i.e., to determine the direction of the described changes, the authors of the study performed a regression in which depression was the dependent variable. We have observed that 11% of the variability in the HADS-D model could be explained by gender and headache, and these variables predisposed them to higher depression scores. Furthermore, headache caused the average HADS-D score to increase by almost 2 points while belonging to the female gender increased this result by an average of 1.69 points. Other authors have reported similar results regarding the higher prevalence of headache in women [34,35,36]. 

The headache during COVID-19 itself did not significantly affect post-COVID quality of life; however, by influencing psychological parameters, it ultimately indirectly influenced its domains as well. The greatest indirect impact on quality of life was recorded for its areas: social and psychological. Our results are consistent with those of Perlis et al. [15] The authors found that individuals with prior COVID-19, who reported headache during acute infection, appeared to have an elevated risk of depressive symptoms. However, as the authors underline, as a cross-sectional study, the possibility that individuals with current depression are more likely to recall or report headache cannot be excluded. 

### 4.1. Limitation

Despite its novelty and many strengths, this study is not without some limitations. This study was carried out in a single center and involved only inpatients, this decreases the ability to generalize the study and, therefore, extrapolates the results to patients with mild forms of the disease, who do not require post-COVID-19 rehabilitation. Additionally, we did not have information on the mental state of the enrolled participants before they contracted COVID-19. Moreover, we did not explore whether the patients suffered of headache or migraine before infection.

### 4.2. Open Questions and Future Research Directions

Although COVID-19 affects primarily physical health, the secondary influence of issues related to the pandemic on mental health should also be considered. Further studies with larger samples, prospective nature with multiple points of clinical data collection and analysis, taking into account inflammatory markers, could help clarify the mechanisms of COVID-19 headache and its association with severity of acute phase of the disease and its long-term consequences. When analyzing the results of this study, numerous questions arose that remain open, such as (1) Did subjects with COVID-19 headache have anxiety-depressive symptoms before COVID-19? (2) Does the presence of anxiety-depressive symptoms prior to COVID-19 predict a more severe course of infection with a severe headache? (3) Is COVID-19 headache and the subsequent presence of anxiety-depressive symptoms related to inflammatory causes? (4) Could the COVID-19 headache be the cause of a new depressive episode? (5) Why does COVID-19 headache occur more frequently in women? Perhaps answering these questions will provide a clearer understanding of the relationship between headaches and the mental state of COVID-19 patients.

## 5. Conclusions

Our study demonstrated several relationships between headaches that occurred during COVID-19 and the emotional state of a patient who underwent post-COVID rehabilitation. Headache during COVID-19 was strongly associated with an increased intercity of depressive-anxiety symptoms and higher stress levels. In turn, psychological parameters were found to be significant mediators of the impact of headache on the quality of life of COVID-19 survivors. Female gender was significantly associated with headache during COVID-19 infection and post-COVID psychological disturbances.

## Figures and Tables

**Table 1:** Characteristics of the participants.

	Total (*n* = 147)	Participants with Headache (*n* = 96)	Participants without Headache (*n* = 51)	*p* Value *
Gender, *n* (%)				
Female	83 (56.46)	66 (68.75)	17 (33.33)	<0.001
Male	64 (43.54)	30 (31.25)	34 (66.67)	
Age, years, mean (SD)	56.97 (9.86)	55.47 (9.65)	59.78 (9.72)	0.01
Body mass, kg, mean (SD)	83.69 (17.07)	82.14 (18.79)	86.59 (12.95)	0.02
Body height, cm, mean (SD)	168.13 (12.41)	167.28 (7.51)	171.67 (10.67)	0.01
BMI, mean (SD)	29.28 (5.12)	29.21 (5.70)	29.42 (3.86)	0.38
Professional activity years, *n* (%)	31.10 (9.08)	29.94 (9.37)	33.33 (8.13)	0.041
Education, *n* (%)				
Basic/vocational	17 (11.56)	9 (9.38)	8 (15.69)	0.10
Secondary	59 (40.14)	34 (35.42)	25 (49.02)	
Higher education	68 (46.26)	50 (52.08)	18 (35.29)	
Missing	3 (2.04)	3 (3.13)	0 (0.00)	
Current employment status, *n* (%)				
Professionally active	96 (65.31)	68 (70.83)	28 (54.90)	0.09
Retirement	42 (28.57)	22 (22.92)	20 (39.22)	
Sickness pension	8 (5.44)	6 (6.25)	2 (3.92)	
Missing	1 (0.68)	0 (0.00)	1 (1.96)	
Marital status, *n* (%)				
Married	104 (70.75)	64 (66.67)	40 (78.43)	0.28
Single	12 (8.16)	10 (10.42)	2 (3.92)	
Divorced	15 (10.20)	12 (12.50)	3 (5.88)	
Widow	11 (7.48)	7 (7.29)	4 (7.84)	
Missing	5 (3.40)	3 (3.13)	2 (3.92)	
Type of profession, *n* (%)				
Intellectual	51 (34.69)	36 (37.50)	15 (29.41)	0.055
Physical	36 (24.49)	18 (18.75)	18 (35.29)	
Mixed	39 (26.53)	29 (30.21)	10 (19.61)	
Missing	21 (14.29)	13 (13.54)	8 (15.69)	
Hypertension, *n* (%)				
Yes	70 (47.62)	37 (38.54)	33 (64.71)	0.002
No	73 (49.66)	56 (58.33)	17 (33.33)	
Missing	4 (2.72)	3 (3.13)	1 (1.96)	
Diabetes, *n* (%)				
Yes	29 (19.73)	18 (18.75)	11 (21.57)	0.64
No	117 (79.59)	78 (81.25)	39 (76.47)	
Missing	1 (0.68)	0 (0.00)	1 (1.96)	
Subjective view of patient health before COVID-19, *n* (%)				
Good	100 (68.03)	62 (64.58)	38 (1.96)	0.48
Neither good nor bad	41 (27.89)	29 (30.21)	12 (23.53)	
Bad	5 (3.40)	4 (4.17)	1 (1.96)	
Missing	1 (0.68)	1 (1.04)	0 (0.00)	
Subjective view of the current patient’s health, *n* (%)				
Good	11 (7.48)	5 (5.21)	6 (11.76)	0.037
Neither good nor bad	99 (67.35)	61 (63.54)	38 (74.51)	
Bad	37 (25.17)	30 (31.25)	7 (13.73)	
Missing	0 (0.00)	0 (0.00)	0 (0.00)	
Physical activity before COVID-19, *n* (%)				
Yes	104 (70.75)	69 (71.88)	35 (68.63)	0.68
No	43 (29.25)	27 (28.13)	16 (31.37)	
Missing	0 (0.00)	0 (0.00)	0 (0.00)	
COVID-19 treatment in, *n* (%)				
Home	65 (44.22)	53 (55.21)	12 (23.53)	<0.001
Hospital	81 (55.10)	42 (43.75)	39 (76.47)	
Missing	1 (0.68)	1 (1.04)	0 (0.00)	
Need for oxygen therapy, *n* (%)				
Yes	81 (55.10)	46 (47.92)	35 (68.63)	0.01
No	66 (44.90)	50 (52.08)	16 (31.37)	
Missing	0 (0.00)	0 (0.00)	0 (0.00)	
Subjective view of the intensity of the COVID-19 symptoms, *n* (%)				
Asymptomatic	2 (1.36)	1 (1.04)	1 (1.96)	0.18
Mild	9 (6.12)	3 (3.13)	6 (11.76)	
Moderate	44 (29.93)	31 (32.29)	13 (25.49)	
Severe	92 (62.59)	61 (63.54)	31 (60.78)	
Missing	0 (0.00)	0 (0.00)	0 (0.00)	
Abdominal pain during COVID-19, *n* (%)				
Yes	37 (25.17)	31 (32.29)	6 (11.76)	0.006
No	110 (74.83)	65 (67.71)	45 (88.24)	
Missing	0 (0.00)	0 (0.00)	0 (0.00)	
Muscle pain during COVID-19, *n* (%)				
Yes	108 (73.47)	82 (85.43)	26 (50.98)	<0.001
No	39 (26.54)	14 (14.58)	25 (49.02)	
Missing	0 (0.00)	0 (0.00)	0 (0.00)	
Loss of smell and taste during COVID-19, *n* (%)				
Yes	90 (61.22)	67 (69.79)	23 (45.10)	0.003
No	57 (38.78)	29 (30.21)	28 (54.90)	
Missing	0 (0.00)	0 (0.00)	0 (0.00)	
Other symptoms, *n* (%)				
None	73 (49.66)	41 (42.71)	32 (62.75)	0.23
Hair loss	3 (2.04)	3 (3.13)	0 (0.00)	
Diarrhea/vomiting	15 (10.20)	12 (12.50)	3 (5.88)	
Dyspnea	16 (10.88)	10 (10.42)	6 (11.76)	
Weakness	18 (12.24)	14 (14.58)	4 (7.84)	
Nonspecific pain	21 (14.29)	15 (15.63)	6 (11.76)	
Skin rash	1 (0.68)	1 (1.04)	0 (0.00)	
Missing	0 (0.00)	0 (0.00)	0 (0.00)	
Persisting pulmonary complications, *n* (%)				
Yes	126 (85.71)	83 (86.46)	43 (84.31)	0.72
No	21 (14.29)	13 (13.54)	8 (15.69)	
Missing	0 (0.00)	0 (0.00)	0 (0.00)	
Persisting cardiac complications, *n* (%)				
Yes	38 (25.85)	27 (28.13)	11 (21.57)	0.39
No	109 (74.15)	69 (71.88)	40 (78.43)	
Missing	0 (0.00)	0 (0.00)	0 (0.00)	
Persisting neurological complications, *n* (%)				
Yes	54 (36.73)	43 (44.79)	11 (21.57)	0.005
No	93 (63.27)	53 (55.21)	40 (78.43)	
Missing	0 (0.00)	0 (0.00)	0 (0.00)	
Persisting mental complications, *n* (%)				
Yes	64 (43.54)	47 (48.96)	17 (33.33)	0.07
No	83 (56.46)	49 (51.04)	34 (66.67)	
Missing	0 (0.00)	0 (0.00)	0 (0.00)	
Persisting other complications, *n* (%)				
Brak	104 (70.75)	68 (70.83)	36 (70.59)	0.60
Hair loss	5 (3.40)	4 (4.17)	1 (1.96)	
Diarrhea/vomiting	0 (0.00)	0 (0.00)	0 (0.00)	
Dyspnea	1 (0.68)	1 (1.04)	0 (0.00)	
Weakness	14 (9.52)	8 (8.33)	6 (11.76)	
Nonspecific pain	16 (10.88)	9 (9.38)	7 (13.73)	
Skin rash	0 (0.00)	0 (0.00)	0 (0.00)	
Memory loss	7 (4.76)	6 (6.25)	1 (1.96)	
Missing	0 (0.00)	0 (0.00)	0 (0.00)	

* *p* Value as a result of chi-squared test (for categorical variables) and unpaired *t*-test (for continues variables).

**Table 2:** Intergroup comparison.

	Participants with Headache (*n* = 96)		Participants without Headache (*n* = 51)		Mean Diff.	*t*	Cohen’s *d*	*p* Value
Variable	Mean	SD	Mean	SD				
HADS total	17.07	8.27	12.90	6.67	4.17	3.33	0.55	0.001
HADS-D	8.90	4.42	6.47	3.54	2.43	3.39	0.61	<0.001
HADS-A	8.18	4.65	6.43	3.87	1.75	2.29	0.41	0.023
PSS-10	20.25	7.59	17.51	6.08	2.74	2.23	0.40	0.028
WHOQOL-BREF domains								
Physical health	19.77	6.78	22.22	3.07	−2.45	−2.44	0.46	0.016
Psychological	19.81	3.23	21.06	2.51	−1.25	−2.40	0.43	0.018
Social relationships	10.97	2.35	11.69	1.53	−0.72	−1.97	0.36	0.051
Environmental	27.80	4.59	30.20	3.94	−2.39	−3.16	0.56	0.002

HADS: Hospital Anxiety (A) and Depression (D) scale; PSS-10: Perceived Stress Scale; *SD*: Standard Deviation.

**Table 3:** Headache, gender, and age as a predictor (stepwise regression results).

Variable	*B*	Beta	*t*	*p* Value	*F*	R^2^
HADS-D				0.01	8.64	0.11
Gender	−1.69	−0.20	−2.34			
Headache	−1.83	−0.20	−2.44			
HADS-A				0.023	5.26	0.04
Headache	−1.75	−0.19	−2.29			
HADS				0.002	6.53	0.07
Gender	−2.48	−0.15	−1.81			
Headache	−3.30	−0.20	−2.33			
PSS-10	−2.74	−0.18	−2.23	0.001	12.28	0.08
Gender	−4.05	−0.28	−3.50			

HADS: Hospital Anxiety (A) and Depression (D) scale; PSS-10: Perceived Stress Scale.

**Table 4:** Results of the mediation analysis.

	Total Effect		Direct Effect		Indirect Effect		Percentage Mediation
Variable	Effect Size (95% CI)	*p* Value	Effect Size (95% CI)	*p* Value	Effect Size (95% CI)	*p* Value	
Physical health	2.44 (0.50, 4.39)	0.01	1.48 (−0.41, 3.37)	0.12	0.96 (0.10, 1.83)	0.03	39.34
Psychological	1.25 (0.23, 2.26)	0.02	0.50 (−0.36, 1.36)	0.26	0.75 (0.12, 1.38)	0.02	60.00
Social relationships	0.72 (0.01, 1.43)	0.04	0.22 (−0.42, 0.87)	0.49	0.49 (0.10, 0.88)	0.01	68.06
Environmental	2.39 (0.92, 3.87)	0.001	1.35 (−0.03, 2.73)	0.05	1.04 (0.29, 1.79)	0.006	43.51

CI: Confidence interval.

## Data Availability

Data is available from the corresponding author upon reasonable request.
